# MicroRNA-505, Suppressed by Oncogenic Long Non-coding RNA LINC01448, Acts as a Novel Suppressor of Glycolysis and Tumor Progression Through Inhibiting HK2 Expression in Pancreatic Cancer

**DOI:** 10.3389/fcell.2020.625056

**Published:** 2021-01-15

**Authors:** Zhenglei Xu, Dingguo Zhang, Zhuliang Zhang, Weixiang Luo, Ruiyue Shi, Jun Yao, Defeng Li, Lisheng Wang, Bihong Liao

**Affiliations:** ^1^The Second Clinical Medical College, Jinan University, Department of Gastroenterology, Shenzhen People's Hospital, Shenzhen, China; ^2^Nursing Department, Shenzhen People's Hospital, The Second Affiliated Clinical Medical College of Jinan University, The First Affiliated Hospital of Southern University of Science and Technology, Shenzhen, China; ^3^Department of Cardiology, Shenzhen People's Hospital, The Second Affiliated Clinical Medical College of Jinan University, The First Affiliated Hospital of Southern University of Science and Technology, Shenzhen, China

**Keywords:** long non-coding RNA, microRNA, miR-505, HK2, pancreatic cancer

## Abstract

**Background:** MicroRNAs (miRNAs) and long non-coding RNAs (lncRNAs) play vital regulatory roles in pancreatic cancer (PC) initiation and progression. We aimed to explore the biological functions and underlying mechanisms of miR-505-3p (miR-505) in PC.

**Methods:** We first screened miRNA expression profiles using microarray in PC tissues and normal tissues, and then studied the function and underlying mechanism of miR-505. Moreover, we evaluated the regulatory effect of lncRNA LINC01448 on miR-505.

**Results:** We demonstrated miR-505 that was significantly downregulated in PC tissues. We further revealed that miR-505 significantly inhibited cell proliferation, invasion, sphere formation, glucose consumption, and lactate production by targeting HK2. In addition, overexpression of miR-505 led to tumor growth inhibition *in vivo*, demonstrating that it acts as a tumor suppressor in PC. LINC01448 was identified as an oncogenic lncRNA that could reduce miR-505 expression. Subsequent studies confirmed that LINC01448 enhanced cell proliferation, invasion, sphere formation, glucose consumption, and lactate production by regulating the miR-505/HK2 pathway.

**Conclusions:** These findings demonstrated that miR-505, suppressed by LINC01448, could function as a key tumor suppressor by targeting HK2 in PC, elucidating an important role of the LINC01448/miR-505/HK2 pathway in regulating PC glycolysis and progression.

## Background

Pancreatic cancer (PC) is one of the most lethal malignancies worldwide (Garrido-Laguna and Hidalgo, 2015). The 5-year overall survival rate of PC has remained low at 6% (Garrido-Laguna and Hidalgo, 2015). PC is usually detected at advanced tumor stages when the disease has metastasized (Avula et al., 2020). Cancer metastasis is the leading cause of PC-related mortality (Sergeant et al., 2009; Avula et al., 2020). Thus, interventions to development and metastasis are critical for a favorable outcome. A growing body of evidence suggested that cancer stem cells constitute a distinct subpopulation in the tumor and play pivotal roles in tumor initiation and progression of PC (Sergeant et al., 2009; Avula et al., 2020). Moreover, most tumor cells highly rely on aerobic glycolysis to produce energy rather than mitochondrial oxidative phosphorylation even in the presence of oxygen, this is known as the “Warburg effect” (Avula et al., 2020). Hexokinase 2 (HK2) is a cancer-associated isoenzyme that catalyzes the first rate-limiting step of glucose metabolism, and depletion of HK2 in PC cell lines decreased lactate production, invasion, and metastasis (Anderson et al., 2016). However, the molecular mechanisms underlying the regulation of PC glycolysis and development are poorly understood.

Long non-coding RNAs (lncRNAs) are a group of RNA transcripts longer than 200 nucleotides without protein-coding potential (Huarte, 2015; Duguang et al., 2017). Recent studies have shown that lncRNAs are involved in the regulation of tumor cell's malignant behavior, including proliferation, invasion, and cancer stemness (Huarte, 2015; Duguang et al., 2017). Although the functions of most lncRNAs are largely unknown, lncRNAs have been shown to have important roles in affecting gene expression at both transcriptional and post-transcriptional levels (Huarte, 2015; Duguang et al., 2017). LncRNAs can function as guides, dynamic scaffolds, molecular decoys, and sponges *via* interaction with components of the cellular machinery, including DNA, RNA, and proteins (Balas and Johnson, 2018; Xu et al., 2020). Moreover, numerous lncRNAs are aberrantly expressed in PC and play essential roles in regulating the tumorigenesis, glycolysis, and metastasis of PC (Huarte, 2015; Duguang et al., 2017; Weidle et al., 2017).

MicroRNAs (miRNAs) are endogenous, small non-coding RNAs that regulate gene expression *via* inhibition of translation or the degradation of mRNA (Chan and Wang, 2015). MiRNAs are dysregulated in almost all types of cancers (including PC), and act as key regulators of cancer development and progression (Chan and Wang, 2015; Dong et al., 2018a). Previous studies have shown that miR-505-3p (miR-505) functions as a tumor suppressor in endometrial cancer (Chen S. et al., 2016), hepatocellular carcinoma (Lu et al., 2016), cervical cancer (Ma et al., 2017), osteosarcoma (Liu et al., 2018), glioma (Shi et al., 2018), and gastric cancer (Tian et al., 2018). Moreover, miR-505 inhibited the IGF-1R/AKT/GLUT-1 pathway and suppressed the glycolysis of hepatocellular carcinoma cells (Ren et al., 2019). Enhanced expression of miR-505 inhibited the expression of an oncogenic lncRNA ZEB1-AS1 in PC cells (Wei et al., 2020). However, the detailed functions and the underlying molecular mechanisms of miR-505 in PC remain unclear.

In this study, we found that miR-505 could significantly inhibit the proliferation, invasion, sphere formation, and glycolysis of PC cells by directly targeting HK2. Our results also revealed that miR-505 suppressed the growth of PC cells *in vivo*. Further mechanistic studies revealed that lncRNA LINC01448 could act as a suppressor of miR-505 to increase HK2 expression. Our findings provided new insights into the role of the LINC01448/miR-505/HK2 axis in PC progression, and this signaling pathway might represent a promising therapeutic target for PC treatment.

## Materials and Methods

### Tissue Specimens

Specimens of PC tissues and corresponding adjacent normal tissues were obtained from 50 patients undergoing surgery at the Shenzhen People's Hospital, The Second Affiliated Clinical Medical College of Jinan University, The First Affiliated Hospital of Southern University of Science and Technology, China. These patients had not received prior treatment before surgery. This research was carried out in accordance with the Helsinki declaration and approved by the Research Ethics Committee of Shenzhen People's Hospital, The Second Affiliated Clinical Medical College of Jinan University, The First Affiliated Hospital of Southern University of Science and Technology. Written informed consent was obtained from each patient. All tissues were immediately frozen in liquid nitrogen and stored at −80°C for RNA extraction.

### miRNA Microarray Analysis

The total RNA from five PC tissues and paired normal tissues was isolated using the TRIzol reagent (Invitrogen, Carlsbad, CA, USA). The expression profile of miRNAs was analyzed using the Agilent Human miRNA expression array (8 × 60K platform, Agilent, Inc., Santa Clara, CA, USA) following the manufacturer's instructions. In brief, 100 ng of total RNA from each specimen was labeled with cyanine 3-pCp (Cy3) using the miRNA Complete Labeling and Hyb kit (Agilent). Then, Cy3-labeled RNAs were hybridized to a miRNA microarray containing 1,887 human miRNAs. The miRNA microarray was scanned using the Agilent G2600D microarray scanner and analyzed using the Agilent Feature Extraction software package (v11.0.1.1).

### Cell Lines and Transfection

Five PC cell lines (AsPC-1, PANC-1, BxPC-3, SW-1990, and PaCa-2) and a non-tumorous pancreatic cell line HPDE6-C7 were purchased from American Type Culture Collection (Manassas, VA, USA). These cells were cultured in RPMI1640 medium (Thermo Fisher Scientific, Waltham, MA, USA) supplemented with 10% fetal bovine serum (FBS, Thermo Fisher Scientific, Waltham, MA, USA) at 37°C in 5% CO_2_.

The expression vectors expressing LINC01448 (or HK2) and the corresponding empty vector were purchased from Genepharma (Shanghai, China). The small-interfering RNAs (siRNAs) against LINC01448 (or HK2), the control siRNA, miR-505 mimic, control mimic, miR-505 inhibitor, and control inhibitor were from Ribobio (Guangzhou, China). Transient transfection was conducted using the Lipofectamine 3000 reagent (Invitrogen, Carlsbad, CA, USA) in accordance with manufacturer's instructions.

### RNA Extraction and Quantitative Real-Time PCR (qRT-PCR) Assays

Total RNA was isolated using the TRIzol reagent (Invitrogen, Carlsbad, CA, USA). The total RNA was then reverse transcribed using the M-MLV Reverse Transcriptase Kit (Invitrogen, Carlsbad, CA, USA) in accordance with manufacturer's protocol. The expression levels of mRNA were quantified with the SYBR Green quantitative PCR kit (Takara, Dalian, China) on a 7300 Real-Time PCR System (Applied Biosystems, Foster City, CA, USA). The primers were purchased from Genepharma (Shanghai, China). *GAPDH* was used as an internal control. The expression level of miR-505 was examined using the mirVanaTM qRT-PCR microRNA Detection Kit (Ambion, Austin, TX, USA) and normalized to U6.

### Western Blotting Analysis

Cells were lysed with RIPA buffer (Beyotime, Beijing, China) containing 1% protease inhibitor cocktail (Selleck, Houston, TX, USA). An equal amount of protein was separated by SDS-polyacrylamide gel electrophoresis and transferred onto a PVDF membrane (Millipore, Bedford, MA, USA). After this, the membranes were incubated with the primary antibodies: HK2 (1:1,000, Cell Signaling, MA, USA) and GAPDH (1:5,000, Santa Cruz, CA, USA). After washing, the blots were incubated with HRP-conjugated secondary antibodies (Santa Cruz, CA, USA). The positive immunoreactivity was detected using the ECL detection system (Amersham Biosciences, Buckinghamshire, UK). GAPDH was used as the loading control.

### Cell Proliferation Assays

Cell proliferation was assessed 72 h after transfection using the CCK-8 assay (Dojindo Laboratories, Kumamoto, Japan) according to the manufacturer's instructions. The absorbance was determined at the wavelength of 450 nm.

### Transwell Invasion Assays

Cell invasion was assessed as previously reported (Dong et al., 2018b). Transfected cells were seeded into the upper chamber of an insert for invasion assays (8-μm pore size, Corning Costar Co, Lowell, CA, USA) with 500 μl serum-free media. The lower chambers were filled with 750 μl medium containing 10% FBS. After an incubation period of 24 h, the invaded cells were stained with Giemsa (Sigma, St. Louis, MO, USA) for 15 min. The number of cells was counted using an Olympus microscope.

### Sphere Formation Assays

Single cells were cultured in serum-free medium supplemented with B27 (1:50; Invitrogen, Carlsbad, CA, USA), 20 ng/ml basic FGF (BD Biosciences, CA, USA) and 20 ng/ml EGF (Sigma, St. Louis, MO, USA). The cells were monitored for 14 days and the cell clusters that had grown to at least 50 μm in diameter were scored as a sphere.

### Glucose Consumption and Lactate Production Assays

Glucose consumption and lactate production were measured using the Glucose Assay Kit-WST and Lactate Assay Kit-WST (Dojindo Laboratories, Kumamoto, Japan) as described previously (Matsuo et al., 2020).

### *In vivo* Tumor Formation Assays

The experimental procedures involving animals were approved by the Shenzhen People's Hospital, The Second Affiliated Clinical Medical College of Jinan University, The First Affiliated Hospital of Southern University of Science and Technology, China. Four-week-old Nude mice (*n* = 6 per group) were obtained from Beijing HFK Bioscience (Beijing, China). PC cells (2 × 10^6^ cells) were subcutaneously injected into the right flank of nude mice. The length and width of tumors were measured every 3 days, and tumor volume was calculated by the following formula: volume = length (mm) × width^2^ (mm^2^)/2. At 21 days after injection, the mice were sacrificed and tumor tissues were excised.

### Luciferase Reporter Assays

The luciferase reporter plasmids containing wild-type (WT) LINC01448, mutant (MUT) LINC01448 with a mutated miR-505 binding site, WT *HK2* 3′-untranslated region (3′-UTR) fragment, and MUT *HK2* 3′-UTR fragment with a mutated miR-505 binding site, were obtained from Ribobio (Guangzhou, China). PC cells were co-transfected with the indicated reporter plasmids, together with miR-505 mimic, miR-505 inhibitor or the respective negative controls, using the Lipofectamine 3000 (Invitrogen, Carlsbad, CA, USA). After 48 h of incubation, the luciferase signal was quantified using a Dual-Luciferase Reporter Assay System (Promega, Madison, WI, USA) according to the manufacturer's directions.

### RNA Immunoprecipitation (RIP) Assays

A Magna RIP RNA-Binding Protein Immunoprecipitation Kit (Millipore, Billerica, MA, USA) was utilized for the RIP assays. PC cells were lysed with RIP-lysis buffer and the cell extract was incubated with magnetic beads conjugated with anti-Ago2 antibody (Millipore, Bedford, MA, USA) or control anti-IgG antibody (Millipore, Bedford, MA, USA). The beads were incubated with proteinase K to remove proteins. Finally, extracted RNAs were subjected to the qRT-PCR analysis.

### Statistical Analysis

Results are presented expressed as the mean ± standard deviation. The Student's *t*-tests, one-way ANOVA tests, Wilcoxon signed-rank tests, χ^2^-tests and Fisher's exact tests were used to compare the mean values among groups. All experiments were done at least three times. *P* < 0.05 was considered statistically significant.

## Results

### Downregulation of miR-505 in PC Tissues and Cell Lines

**Figure 1:**
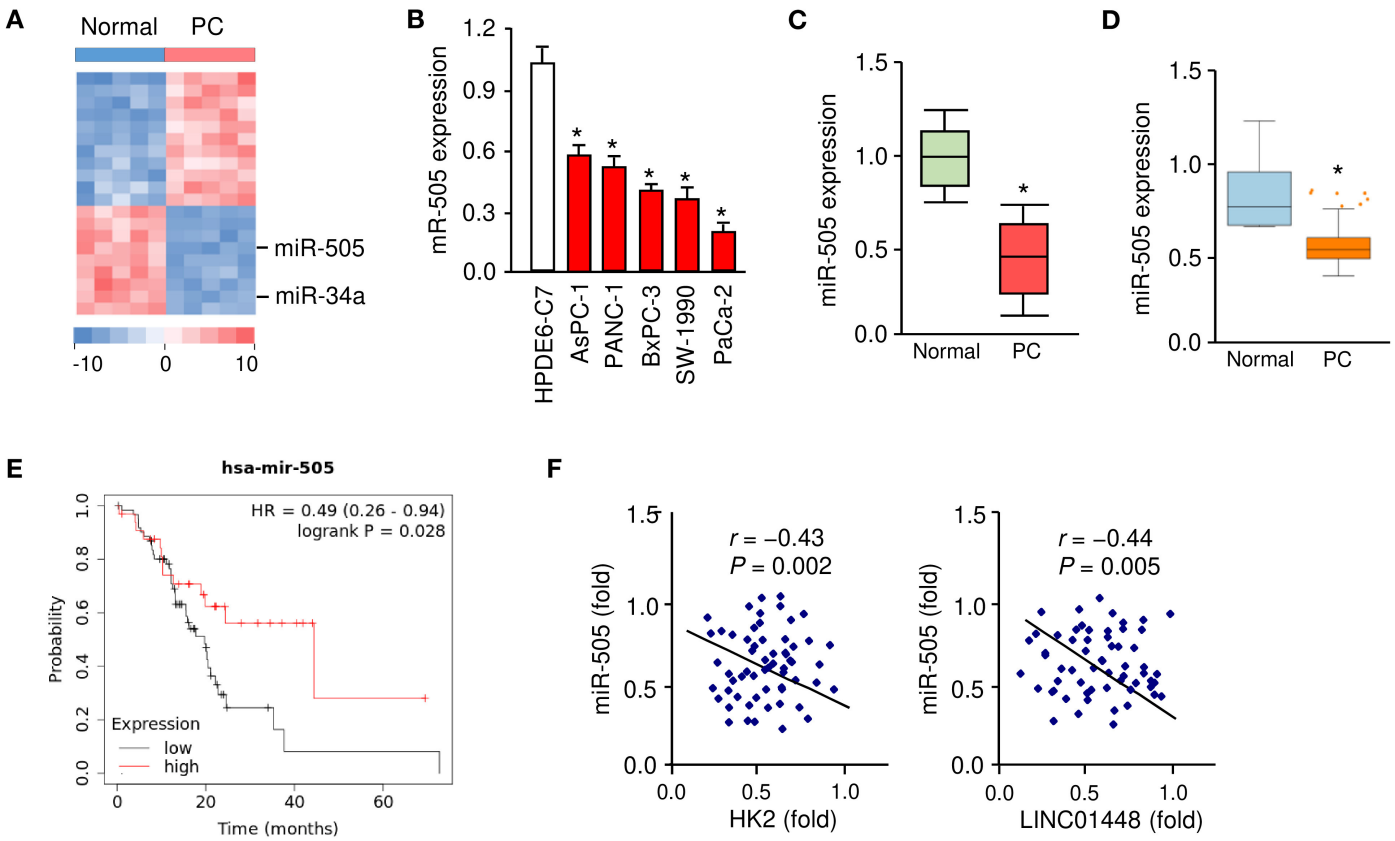
Downregulation of miR-505 in PC tissues and cell lines. **(A)** The differentially expressed miRNAs between five PC tissues and their adjacent normal tissues were identified using a miRNA microarray analysis. **(B)** qRT-PCR analysis of miR-505 expression in PC cell lines and a normal pancreatic cell line HPDE6-C7. **(C)** miR-505 levels in PC tissues and normal tissues were examined using qRT-PCR assays. **(D)** Meta-profiling of differential expression of miR-505 in PC tissues and normal tissues (MIR-TV database). **(E)** Kaplan-Meier curves revealed that lower expression of miR-505 was related to poor overall survival in PC patients (KM Plotter database). **(F)** Correlative analysis of miR-505 and HK2/LINC01448 expression in PC tissues. *P < 0.05.

We applied miRNA microarray analysis to identify the miRNAs that are differentially expressed in PC tissues (*n* = 5) and their adjacent normal tissues (*n* = 5). MiR-34a is a known tumor suppressor downregulated in several types of malignancies including PC (Tang et al., 2017). As expected, miR-34a expression was significantly downregulated in PC samples compared with paired non-cancerous tissues (Figure 1A). We found that miR-505 was the most significantly downregulated miRNA in PC tissues (Figure 1A).

Our qRT-PCR assays revealed that the expression of miR-505 was significantly lower in the PC cell lines, whereas a normal pancreatic duct epithelial cell line HPDE6-C7 expressed a relatively higher expression level of miR-505 (Figure 1B). Furthermore, the qRT-PCR assays were used to determine the expression levels of miR-505 in 50 specimens of PC and normal tissues. miR-505 levels were significantly lower in PC tissues (Figure 1C). In addition, we analyzed the expression of miR-505 across different normal and cancer tissues using the MIR-TV database (http://mirtv.ibms.sinica.edu.tw/index.php). Compared with normal tissues, miR-505 was markedly downregulated in PC tissues (Figure 1D). We then accessed the prognostic value of miR-505 expression in the TCGA PC dataset using the KM Plotter database (http://kmplot.com/analysis/). Survival curves were plotted for PC patients (*n* = 178). High expression of miR-505 was positively associated with a favorable prognosis in PC patients (Figure 1E).

To further define the clinical significance of miR-505 expression in PC, we divided 50 PC tissues into two groups: cancers with below-median miR-505 expression and cancers with above-median miR-505 expression. The correlation of miR-505 expression with various clinical parameters was shown in Table 1. No significant association was found between miR-505 expression and age, tumor differentiation, or tumor size. Interestingly, lower miR-505 levels were significantly correlated with more advanced pathological stage and more lymph node metastasis (Table 1). Together, these results suggested that miR-505 might play a tumor suppressor role in PC.

**Table 1:** Correlation of miR-505 expression with clinical characteristics of patients with PC.

**Characteristics**	**miR-505 expression**		***P* values**
	**Below median**	**Above median**	
**Age (years)**			
>60	14	11	0.778
≤ 60	12	13	
**Differentiation**			
Well/moderate	11	14	0.396
Poor	15	10	
**Tumor size**			
≤ 2 cm	15	16	0.57
>2 cm	11	8	
**TNM stage**			
I/II	5	16	0.0012
III/IV	21	8	
**Lymph node metastasis**			
Negative	4	21	0.0001
Positive	22	3	

### miR-505 Inhibits the Proliferation, Invasion, Sphere Formation, and Glycolysis of PC Cells

**Figure 2:**
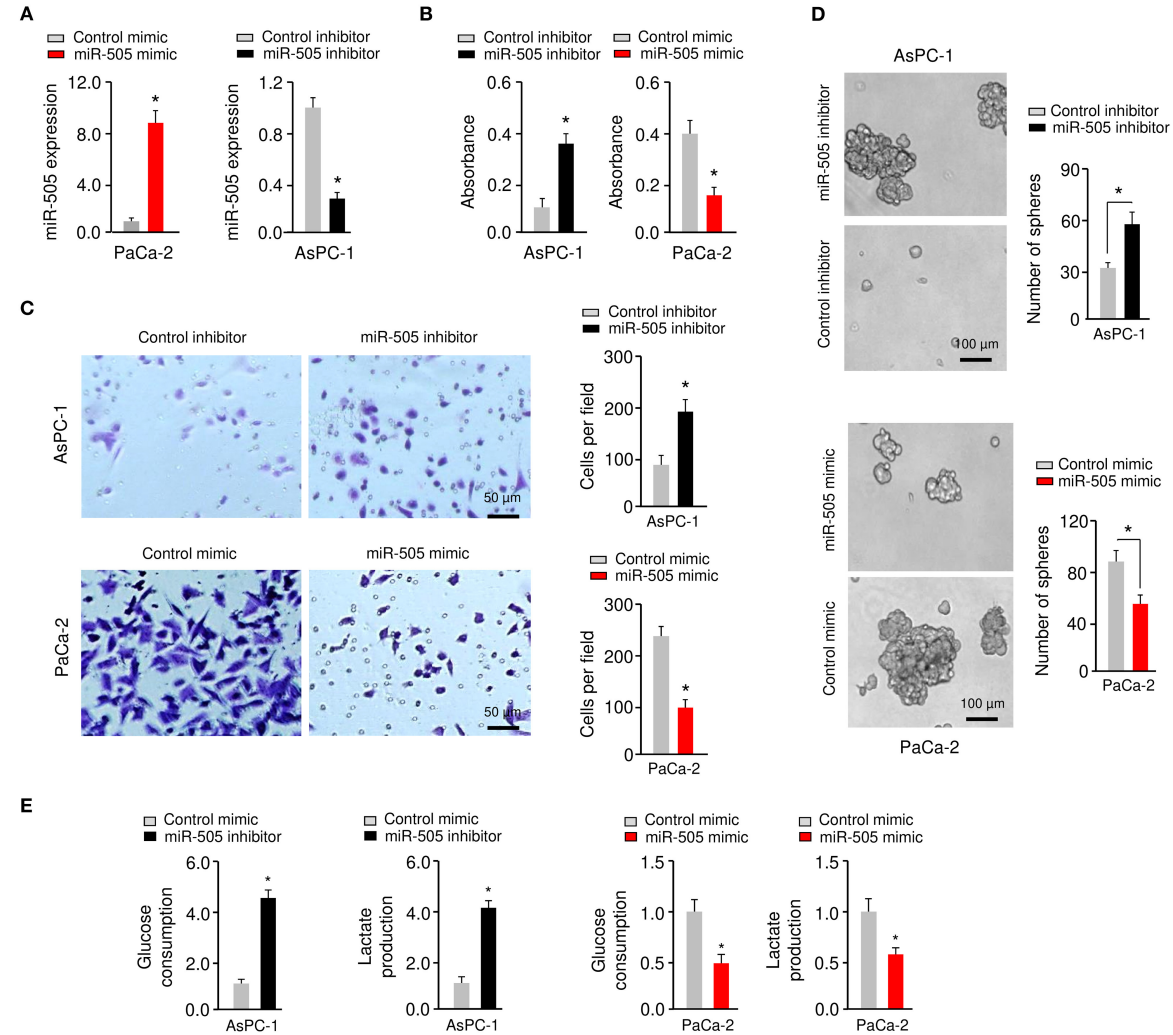
miR-505 inhibits the proliferation, invasion, sphere formation, and glycolysis of PC cells. **(A)** Expression of miR-505 in PC cells transfected with miR-505 mimic, miR-505 inhibitor or their negative controls, respectively. **(B)** Cell proliferation was analyzed using CCK-8 assays. **(C)** Invasion of PaCa-2 (upper) cells was examined after miR-505 overexpression. Invasion of AsPC-1 (bottom) cells was examined after knockdown of miR-505. Scale bar: 50 μm. **(D)** PC cells were transfected as indicated, and sphere formation assays were conducted. Scale bar: 100 μm. **(E)** Glucose consumption and lactate production assays in PaCa-2 cells after overexpression of miR-505, and in AsPC-1 cells after knockdown of miR-505. *P < 0.05.

To determine whether miR-505 could affect the malignant phenotypes in PC cells, we examined the effects of either miR-505 overexpression or knockdown on cell proliferation, invasion, sphere formation, and glycolysis. AsPC-1/PaCa-2 cells with relatively higher/lower miR-505 expression were used in subsequent functional assays (Figure 2A). The overexpression of miR-505 significantly suppressed the proliferation, invasion, sphere formation, glucose consumption, and lactate production of PaCa-2 cells (Figures 2A–E). We also transfected AsPC-1 cells that exhibit higher levels of miR-505 with miR-505 inhibitor and assessed the effects of miR-505 depletion on these malignant properties (Figure 2A). Cell proliferation, invasion, sphere formation, glucose consumption, and lactate production assays demonstrated that knockdown of miR-505 significantly induced the proliferation, invasion, sphere formation, glucose consumption, and lactate production of AsPC-1 cells (Figures 2A–E). Collectively, these data revealed that miR-505 overexpression significantly inhibits the proliferation, invasion, sphere formation, and glycolysis of PC cells.

### miR-505 Represses Tumorigenesis of PC Cells *in vivo*

**Figure 3:**
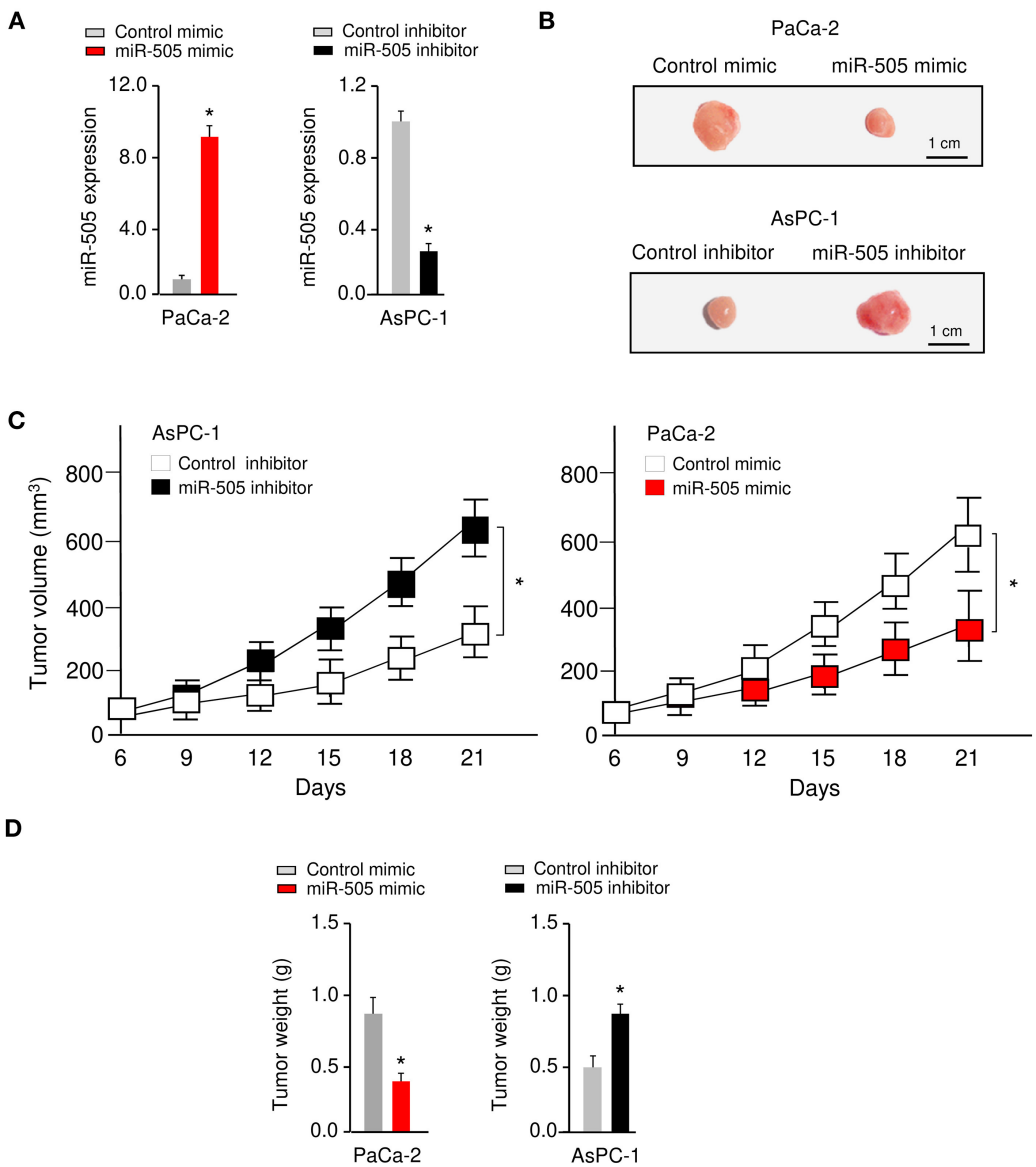
miR-505 represses tumorigenesis of PC cells *in vivo*. **(A)** The levels of miR-505 in PC cells transfected with miR-505 mimic, miR-505 inhibitor or their negative controls, respectively. **(B–D)** Nude mouse xenograft model**s** were established by subcutaneously implanting PaCa-2 cells transfected with (or without) miR-505 mimic, or AsPC-1 cells transfected with (or without) miR-505 inhibitor, respectively. Representative images **(B)**, tumor volume growth curves **(C)**, and weight **(D)** of the formed tumors. Scale bar: 1 cm. *P < 0.05.

To further investigate the functional role of miR-505 in PC *in vivo*, we established nude mouse xenograft models by implanting PaCa-2 cells transfected with miR-505 mimic (or control mimic), or AsPC-1 cells transfected with miR-505 inhibitor (or control inhibitor), respectively (Figure 3A). The tumor growth was monitored, and we found that overexpression of miR-505 significantly decreased the tumor volumes and tumor weights, whereas silencing of miR-505 significantly enhanced the tumor volumes and tumor weights (Figures 3B–D). Taken together, these results demonstrated that miR-505 inhibits the tumorigenesis of PC cells *in vivo*.

### miR-505 Directly Targets HK2 in PC Cells

**Figure 4:**
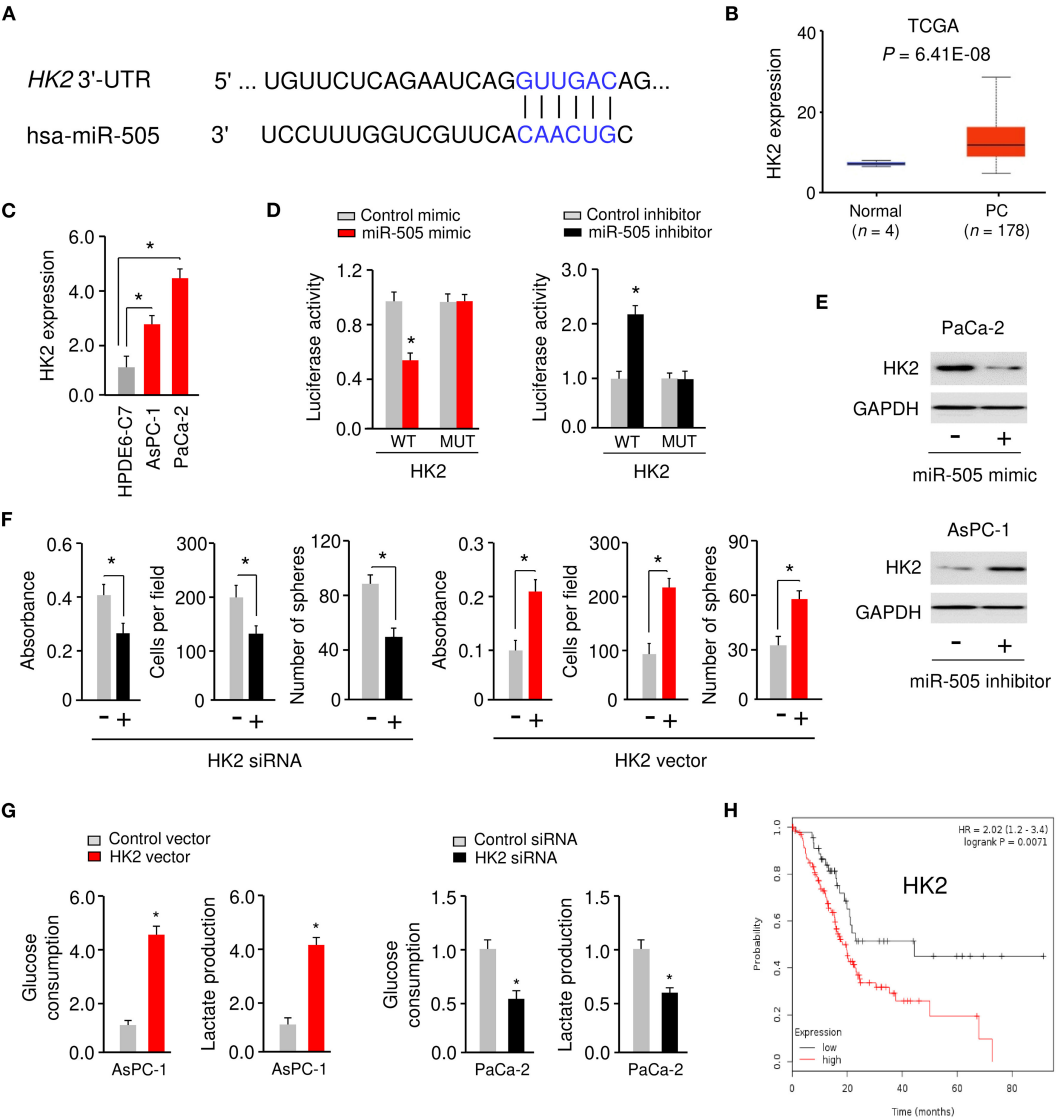
miR-505 directly targets HK2 in PC cells. **(A)** The predicted miR-505 binding site in the *HK2* 3′-UTR sequence. **(B)** The expression of HK2 in PC tissues and normal tissues (UALCAN database). **(C)** qRT-PCR analysis of HK2 expression in PC cell lines and normal pancreatic cells. **(D)** PaCa-2 cells were transfected with luciferase reporter vectors containing wild-type (WT) or mutant (MUT) *HK2* 3′-UTR, along with (or without) miR-505 mimic, and AsPC-1 cells were transfected with luciferase reporter vectors containing WT or MUT *HK2* 3′-UTR, along with (or without) miR-505 inhibitor. The binding between miR-505 and HK2 was verified using luciferase reporter assays. **(E)** Western blotting analysis of HK2 expression in PaCa-2 cells transfected with (or without) miR-505 mimic, and in AsPC-1 cells transfected with (or without) miR-505 inhibitor. **(F)** PC cells were transfected with HK2 vector or HK2 siRNA as indicated, and cell proliferation, invasion and sphere formation assays were performed. **(G)** Glucose consumption and lactate production assays in PaCa-2 cells after knockdown of HK2 and in AsPC-1 cells after overexpression of HK2. **(H)** Kaplan-Meier curves for the overall survival of PC patients were compared between groups with high or low levels of HK2 based on the TCGA data from the KM Plotter database. *P < 0.05.

We predicted the possible target genes of miR-505 using the TargetScan database (http://www.targetscan.org). According to this analysis, miR-505 could target the 3′-UTR of *HK2* mRNA (Figure 4A). To explore the potential role of HK2 in PC, we searched the UALCAN database (http://ualcan.path.uab.edu/index.html) for *HK2* expression in the TCGA PC tissues. This analysis revealed the significant upregulation of *HK2* in PC tissues compared with normal tissues (Figure 4B). Then, we performed qRT-PCR assays to analyze the level of *HK2* in PC cell lines (AsPC-1 and PaCa-2) and HPDE6-C7 cells. We found that *HK2* was overexpressed in PC cells compared with HPDE6-C7 cells (Figure 4C).

To analyze whether miR-505 directly represses HK2 expression by binding to its 3′-UTR, we performed the luciferase reporter assays by co-transfecting a reporter vector containing WT *HK2* 3′-UTR with miR-505 mimic (or miR-505 inhibitor) into PC cells. Transfection with miR-505 mimic significantly inhibited *HK2* luciferase activity in PaCa-2 cells, whereas transfection with miR-505 inhibitor significantly increased *HK2* luciferase activity in AsPC-1 cells (Figure 4D). However, transfection of miR-505 mimic or miR-505 inhibitor did not significantly alter the luciferase reporter activity of mutated *HK2* 3′-UTR (Figure 4D). Western blotting analysis confirmed that overexpression of miR-505 led to downregulation of HK2 in PaCa-2 cells (Figure 4E). On the contrary, knockdown of miR-505 increased HK2 protein levels in AsPC-1 cells (Figure 4E). These results verified that *HK2* is a target gene of miR-505.

We examined the effects of HK2 expression on PC cell proliferation, invasion, sphere formation, and glycolysis. Our cell functional assays suggested that overexpression of HK2 significantly induced the proliferation, invasion, sphere formation, glucose consumption, and lactate production of AsPC-1 cells (Figures 4F,G). However, silencing of HK2 significantly impaired the proliferation, invasion, sphere formation, glucose consumption, and lactate production of PaCa-2 cells (Figures 4F,G). Furthermore, Kaplan-Meier survival analysis using data from the KM Plotter database showed that those PC patients with high HK2 expression had worse overall survival (Figure 4H). These results have identified HK2 as a novel oncogene in PC cells and suggested that miR-505 negatively regulates the expression of HK2.

### miR-505 Acts as a Tumor Suppressor by Inhibiting HK2 Expression in PC Cells

**Figure 5:**
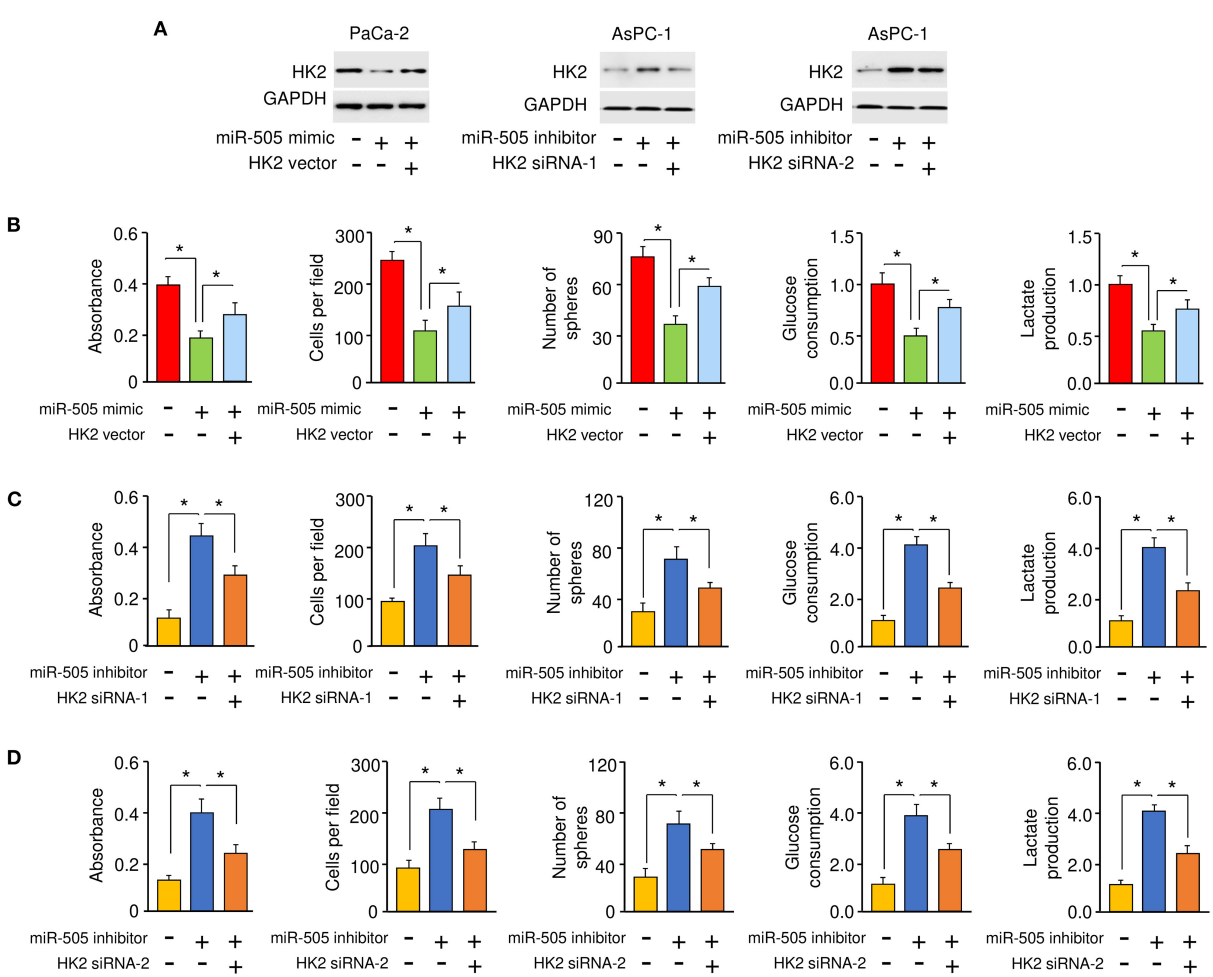
miR-505 acts as a tumor suppressor by inhibiting HK2 expression in PC cells. **(A)** Western blotting analysis of HK2 expression in PaCa-2 cells transfected with or without miR-505 mimic, together with (or without) HK2 vector, or in AsPC-1 cells transfected with or without miR-505 inhibitor, together with (or without) HK2 siRNAs. **(B)** Cell functional assays were conducted in PaCa-2 cells transfected with (or without) miR-505 mimic, along with (or without) HK2 expression vector. **(C,D)** Cell functional assays were done in AsPC-1 cells transfected with (or without) miR-505 inhibitor, together with (or without) HK2 siRNA-1 **(C)** or HK2 siRNA-2 **(D)**. *P < 0.05.

To explore whether miR-505 exerts its tumor suppressor functions through suppressing the expression of HK2. PaCa-2cells were co-transfected with miR-505 mimic (or control mimic), together with (or without) HK2 expression vector (Figure 5A). On the other hand, AsPC-1 cells were co-transfected with miR-505 inhibitor (or control inhibitor), together with (or without) HK2 siRNAs, respectively (Figure 5A). Our cell functional assays showed that overexpression of HK2 could restore miR-505 mimic-suppressed PC cell proliferation, invasion, sphere formation, and glycolysis (Figure 5B). In contrast, HK2 siRNA could prevent miR-505 knockdown-enhanced PC cell proliferation, invasion, sphere formation, and glycolysis (Figures 5C,D). Together, our data supported that miR-505 suppresses the malignant properties of PC cells by inhibiting HK2 expression.

### lncRNA LINC01448 Directly Binds to miR-505 and Represses Its Expression

**Figure 6:**
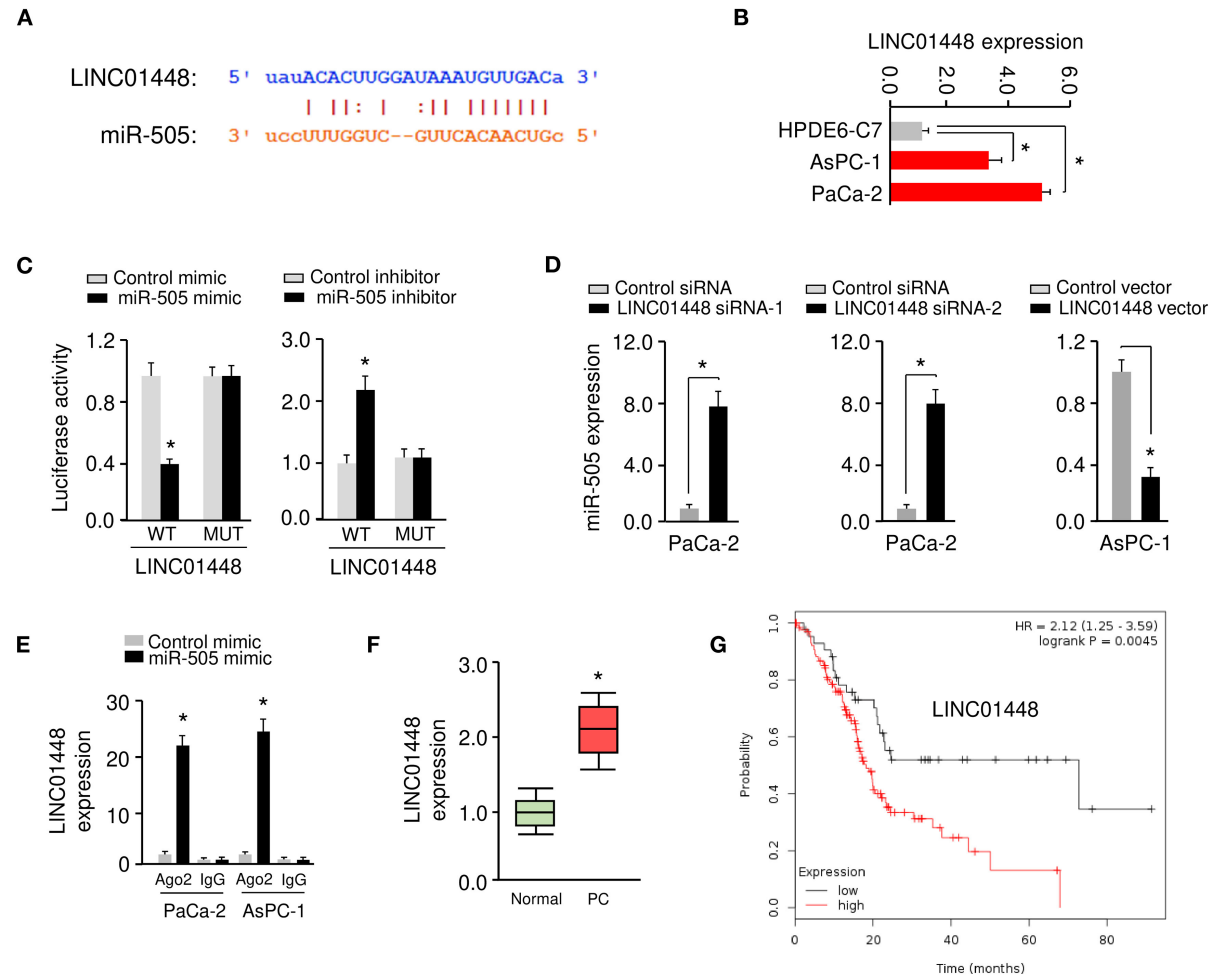
LncRNA LINC01448 directly binds to miR-505 and represses its expression. **(A)** Putative binding sites between miR-505 and LINC01448. **(B)** qRT-PCR analysis of LINC01448 expression in PC cell lines and normal pancreatic cells using. **(C)** Luciferase activity in PaCa-2 cells transfected with WT or MUT LINC01448, along with (or without) miR-505 mimic, or in AsPC-1 cells transfected with WT or MUT LINC01448, along with (or without) miR-505 inhibitor. **(D)** miR-505 expression in PC cells after LINC01448 knockdown or overexpression of LINC01448. **(E)** RIP assays were conducted in PC cells transfected with control mimic and miR-505 mimic. LINC01448 expression was analyzed using qRT-PCR analysis. **(F)** qRT-PCR analysis of LINC01448 expression in PC tissues and normal tissues. **(G)** Kaplan-Meier curves for the overall survival of PC patients with high or low LINC01448 expression (KM Plotter database). *P < 0.05.

Many lncRNAs were known to function as miRNA sponges, leading to the reduction of miRNA expression in tumor cells (Huarte, 2015). Using the NOCORI prediction program (http://starbase.sysu.edu.cn), lncRNA LINC01448 was found to contain one potential binding site for miR-505 (Figure 6A). Thus, we selected the lncRNA LINC01448 for our subsequent study.

The levels of LINC01448 were significantly increased in AsPC-1 and PaCa-2 cells when compared to HPDE6-C7 cells (Figure 6B). To assess the association between LINC01448 and miR-505, we performed the luciferase reporter assays. Overexpression of miR-505 suppressed the luciferase activities of WT LINC01448 reporter constructs, but this effect was abolished when the binding site for miR-505 within the LINC01448 sequence was mutated (Figure 6C). We further observed that the inhibition of miR-505 significantly increased the luciferase activities of WT LINC01448, but had no significant effect on the luciferase activities of MUT LINC01448 (Figure 6C). The qRT-PCR experiments suggested that silencing of LINC01448 in PaCa-2 cells led to a significant increase in miR-505 expression, while the overexpression of LINC01448 in AsPC-1 cells significantly reduced the levels of miR-505 (Figure 6D). We conducted RIP assays in PC cells that were transfected with miR-505 mimic or control mimic. Compared with the control group, the endogenous LINC01448 was efficiently pulled down by Ago2 in PC cells transfected with miR-505 mimic (Figure 6E).

Using qRT-PCR assays, we examined the expression of LINC01448 in PC tissues and adjacent normal tissues and found that the expression of LINC01448 was significantly increased in PC tissues (Figure 6F). Then, we explored the correlation between LINC01448 expression and PC patient survival using the KM Plotter database. PC patients were divided into high and low LINC01448 expression groups according to the median value. Kaplan–Meier survival analysis suggested that PC patients with low expression of LINC01448 displayed longer overall survival times than those with high expression of LINC01448 (Figure 6G). Collectively, these results demonstrated that LINC01448 may serve as a sponge for miR-505.

### LINC01448 Promotes the Malignant Phenotypes of PC Cells *via* Repressing miR-505 Expression

**Figure 7:**
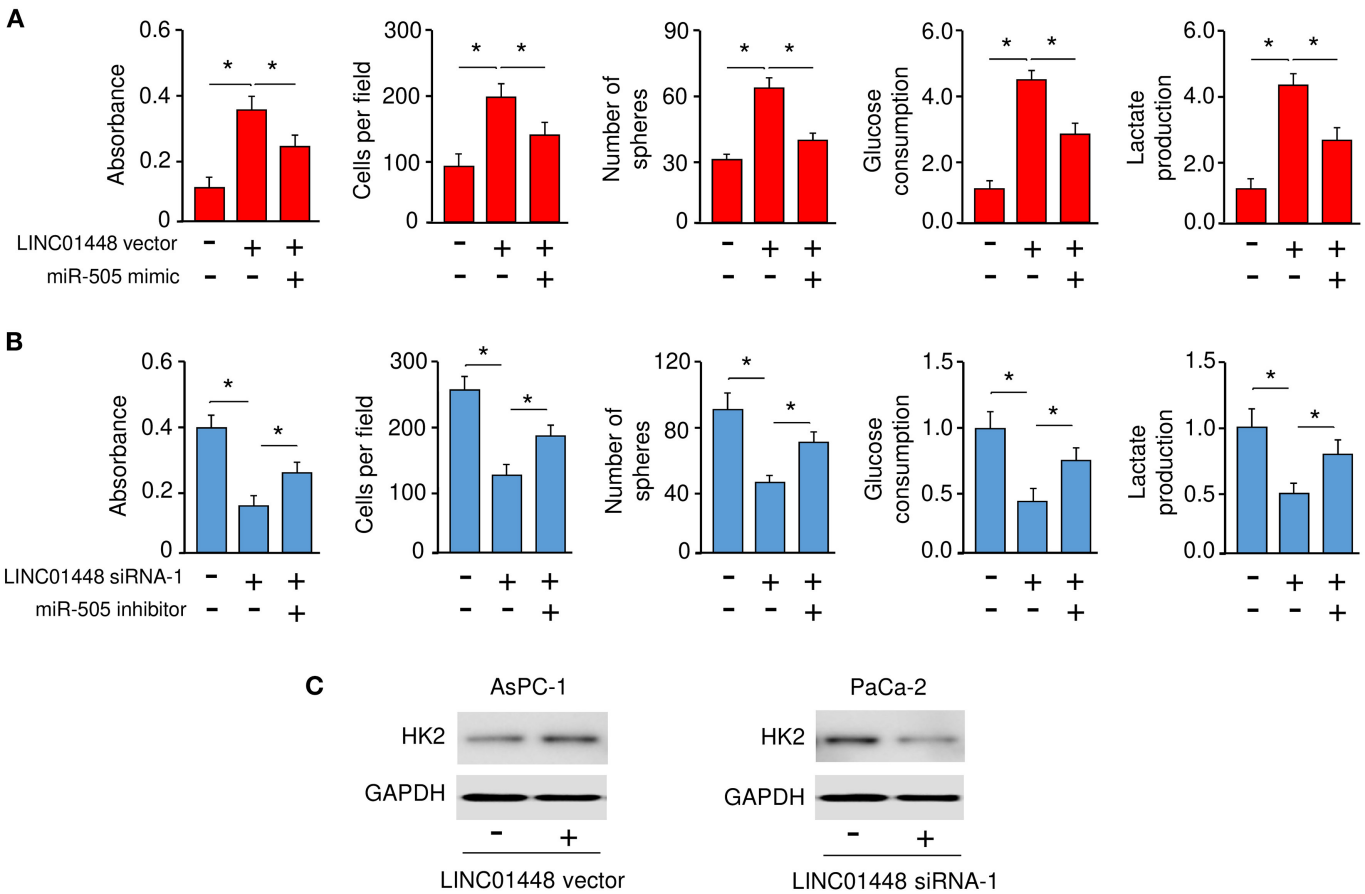
LINC01448 promotes cell proliferation, invasion, sphere formation, and glycolysis by repressing miR-505 expression. **(A)** Cell functional assays were evaluated in AsPC-1 cells transfected with (or without) the LINC01448 expression vector, along with (or without) miR-505 mimic. **(B)** Cell functional assays in PaCa-2 cells transfected with (or without) LINC01448 siRNA-1, along with (or without) miR-505 inhibitor. **(C)** Western blotting analysis of HK2 expression in PaCa-2 cells transfected with (or without) LINC01448 siRNA-1, and in AsPC-1 cells transfected with (or without) LINC01448 expression vector. *P < 0.05.

To investigate whether LINC01448 modulates the malignant phenotypes of PC cells through repressing miR-505 expression, we conducted rescue experiments. AsPC-1 cells were transfected with a LINC01448 expression vector (or control vector), together with (or without) miR-505 mimic. Conversely, PaCa-2 cells were transfected with LINC01448 siRNA (or control siRNA), together with (or without) miR-505 inhibitor. Our cell functional assays showed that the overexpression of LINC01448 promoted the proliferation, invasion, sphere formation, glucose consumption, and lactate production of AsPC-1 cells, and these effects were partially reversed by the restoration of miR-505 expression (Figure 7A). Furthermore, the inhibition of miR-505 partially reversed the LINC01448 siRNA-reduced cell proliferation, invasion, sphere formation, glucose consumption, and lactate production (Figure 7B). The protein expression of HK2 was increased in LINC01448-overexpressing AsPC-1 cells, but was reduced in LINC01448-knockdown PaCa-2 cells (Figure 7C). Using qRT-PCR assays, we detected a significant negative association between miR-505 and *HK2*/LINC01448 expression in PC tissues (Figure 1F).

**Figure 8:**
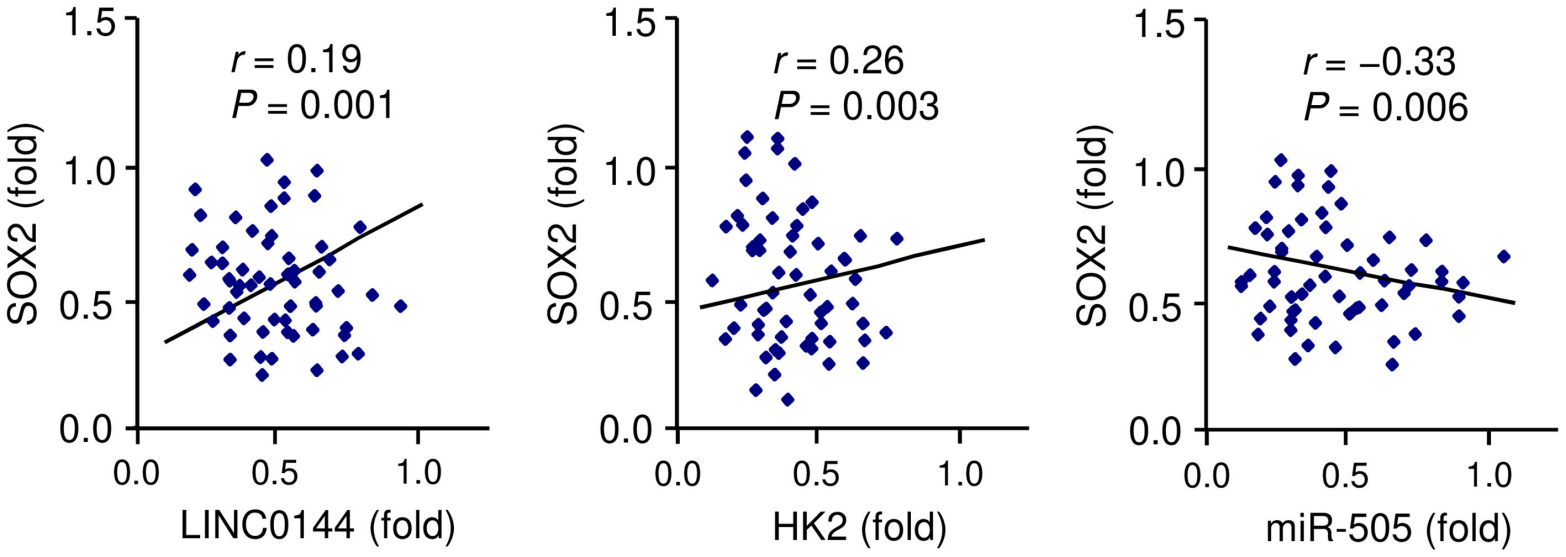
Correlation between SOX2 and LINC01448/miR-505/HK2 expression in PC tissues, as determined by qRT-PCR assays.

To further elucidate the mechanism underlying LINC01448 overexpression in PC, we investigated the involvement of transcription factors in regulating the expression of LINC01448. We used the UCSC database (http://genome.ucsc.edu/) to find possible transcription factors for LINC01448. Among these transcription factors, SOX2 has been recognized as a powerful oncogene in PC (Herreros-Villanueva et al., 2013), and it was reported as a transcription activator of lncRNAs (Wu et al., 2018). Consistent with this prediction, our qRT-PCR analysis showed that SOX2 expression was positively correlated with the levels of LINC01448 and HK2 in PC tissues (Figure 8). In contrast, the expression of SOX2 was negatively correlated with the expression of miR-505 in patients with PC (Figure 8). Taken together, these findings indicated a possibility that SOX2 might act as a transcription activator of LINC01448 in PC.

## Discussion

**Figure 9:**
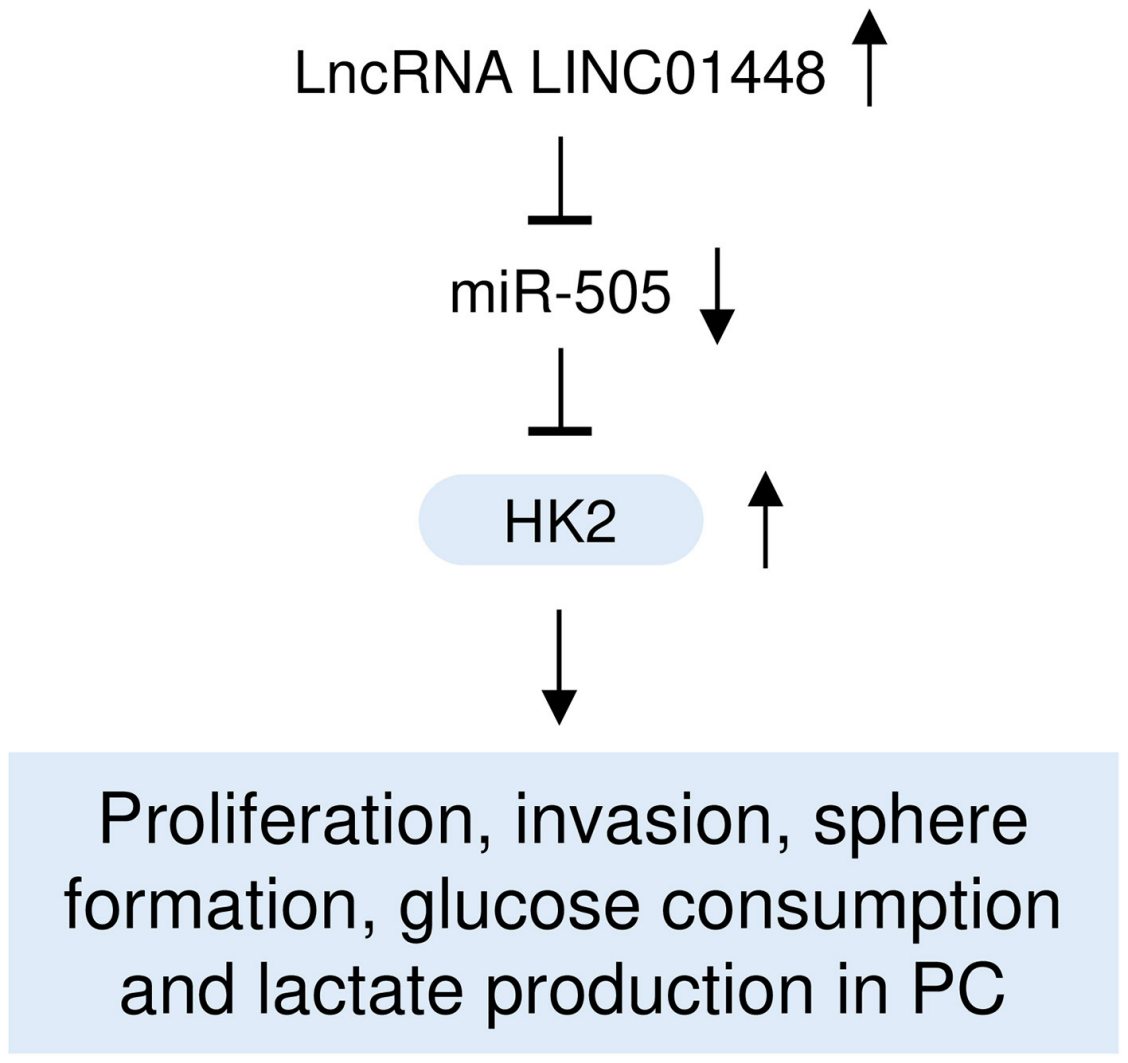
A hypothetical model illustrating that the LINC01448/miR-505/HK2 axis modulates PC glycolysis and progression.

Although several studies have demonstrated that dysregulated miRNAs and lncRNAs could function as important regulators in PC through modulating cancer cell proliferation, migration, and invasion (Zhai et al., 2020), it remains unclear whether miR-505 and LINC00261 regulate the glycolysis and aggressive features of PC cells. To the best of our knowledge, for the first time, this study demonstrated that miR-505 functions as a tumor suppressor to attenuate glycolysis and aggressive phenotypes of PC cells through targeting HK2, and that oncogenic LINC01448 serves as an upstream inhibitor of miR-505 in PC cells (Figure 9).

In this study, we detected differentially expressed miRNAs in PC tissues. Of these miRNAs, miR-505 has been characterized as a tumor-suppressive miRNA in human endometrial cancer (Chen S. et al., 2016), hepatocellular carcinoma (Lu et al., 2016), cervical cancer (Ma et al., 2017), osteosarcoma (Liu et al., 2018), glioma (Shi et al., 2018), and gastric cancer (Tian et al., 2018). Very recently, one study showed that miR-505 inhibited the expression of an oncogenic lncRNA ZEB1-AS1 in PC cells (Ren et al., 2019), however the cellular role of miR-505 in the initiation and progression of PC remain to be elucidated. Here, our *in vitro* and *in vivo* experiments provided the first evidence that miR-505 can play a critical role in suppressing the glycolysis and progression of PC. The expression of miR-505 in PC tissues was significantly lower than that in corresponding normal tissues. Furthermore, the levels of miR-505 were positively associated with the favorable survival of patients with PC. These data suggested that miR-505 might be an effective prognostic biomarker and therapeutic target for PC patients.

A large number of oncogenes as target genes of miR-505 in tumor cells, including TGF-α (Chen S. et al., 2016), HMGB1 (Lu et al., 2016; Liu et al., 2018; Tian et al., 2018), FZD4 (Ma et al., 2017), IGF1R (Shi et al., 2018), WNT7B (Zhang et al., 2018), MAP3K3 (Tang et al., 2019), NRCAM (Ling et al., 2019), and RUNX2 (Zhao et al., 2019), have been recognized as direct target genes of miR-505. Among them, HMGB1 was found to promote cancer progression and metastasis in different cancers, such as hepatocellular carcinoma, lung cancer, breast cancer, colorectal cancer, prostate cancer, cervical cancer, and ovarian cancer (Tripathi et al., 2019). In this study, we proved that miR-505 regulated HK2 expression in PC cells at the post-transcriptional level. It would be possible that the restoration of miR-505 could effectively repress glycolysis and metastasis of PC by targeting other novel oncogenes. Further studies would be needed to explore this possibility.

Tumor cells are known for their increased glucose uptake rates even in the presence of abundant oxygen, defined as the Warburg effect (Avula et al., 2020). HK2 catalyzes the first committed step of glucose metabolism and is critically important for aerobic glycolysis in multiple cancer types (Stolfi et al., 2013). Moreover, knockdown of HK2 in ovarian cancer cells attenuated lactate production, cell invasion, and cancer cell stemness (Siu et al., 2019). Therefore, HK2 has been proposed as a therapeutic target for cancers (Chen X. S. et al., 2016; Garcia et al., 2019). In PC tissues, the expression of HK2 was significantly up-regulated and increased HK2 expression was associated with shorter overall survival in patients with PC (Anderson et al., 2016). Furthermore, upregulation of HK2 can stimulate glycolysis and increase the proliferative and metastatic potential of PC cells (Anderson et al., 2016). Consistent with these previous findings, our current data verified that HK2 promoted glycolysis and facilitated the proliferation, invasion, and cancer stem cell-like properties of PC cells. In this study, we provided the first functional link between miR-505 and HK2, and have defined a new mechanism that drives overexpression of HK2 in PC. Previous papers reported that HK2 was a target of miR-143 (Peschiaroli et al., 2013), miR-199a-5p (Guo et al., 2015), miR-181b (Li et al., 2016), and miR-98 (Zhu et al., 2017). However, it remains largely unknown whether these miRNAs regulate HK2 expression in PC cells.

LncRNAs can act as sponges for miRNAs and thereby negatively regulate their expression (Dong et al., 2018c). For instance, lncRNA-CTS could function as a competing endogenous RNA for miR-505 in cervical cancer cells (Feng et al., 2019). LncRNA LEF-AS1 binds directly to miR-505 and suppressed its expression in colorectal cancer cells (Gong and Huang, 2019). Similarly, LINC00525 acted as a sponge for miR-505 in chordoma cells (Li et al., 2020). Up to now, the role and underlying mechanisms of LINC01448 in PC have not been previously reported. Our bioinformatic analysis, luciferase assay and RIP assay validated a direct interaction between miR-505 and LINC01448, identifying LINC01448 as a key upstream suppressor of miR-505 in PC cells. Our results account, at least in part, for the reduction of miR-505 expression observed in PC tissues. Importantly, we found that LINC01448 sponged miR-505 to facilitate the proliferation, invasion, sphere formation, and glycolysis of PC cells. Thus, our studies supported the notion that the therapeutic inhibition of LINC01448 may serve as a potential therapeutic strategy for PC treatment. Whether or how LINC01448 regulates the expression of other miRNAs in PC cells requires further investigation.

Many researchers have recognized that lncRNAs not only work as miRNA sponges, but also govern basic cellular processes by interacting with RNA-binding proteins (Huarte, 2015; Duguang et al., 2017). A recent study discovered that lncRNA LINC00261 exerted its function in PC cells, by binding to miR-222-3p to activate the HIPK2 pathway and by sequestering an RNA-binding protein IGF2BP1 to reduce c-Myc expression (Zhai et al., 2020). Whether LINC01448 could promote PC development by interacting with RNA-binding proteins should be investigated in our future research.

Multiple studies have shown that transcription factor, DNA methylation and EZH2-mediated H3K27 trimethylation represent important mechanisms that mediate the expression of miRNAs and lncRNAs (Lin et al., 2019; Zhai et al., 2020). For instance, downregulation of LINC00261 was caused by hypermethylation of the CpG islands in its promoter region and EZH2-mediated histone H3 lysine 27 trimethylation in PC cells (Zhai et al., 2020). In addition, p53, the most studied tumor suppressor, was shown to induce the expression of the lncRNA Pvt1b, leading to the suppression of lung tumorigenesis (Olivero et al., 2020). The transcription factor SOX2 induces the expression of lncRNA ANRIL by promoting its transcription in nasopharyngeal carcinoma cells (Wu et al., 2018). Interestingly, SOX2 might bind to the promoter region of LINC01448, and we have detected a positive correlation between the expression of SOX2 and LINC01448/HK2 expression, and an inverse correlation between the levels of SOX2 and miR-505 in PC tissues. Further studies will be needed to elucidate the possibility that SOX2 regulates the levels of LINC01448 in PC cells.

In summary, we found that miR-505 acted as a key tumor suppressor miRNA through targeting oncogene HK2 in PC cells. Our study further revealed that aberrant expression of LINC01448 could reduce the expression of miR-505 to promote proliferation, invasion, sphere formation, and glycolysis in PC cells. These findings provided new insights into the mechanisms that control glycolysis and aggressive phenotypes in PC, and highlighted LINC01448, miR-505 and HK2 as potential targets for therapeutic intervention in patients with PC.

## Data Availability Statement

The raw data supporting the conclusions of this article will be made available by the authors, without undue reservation.

## Ethics Statement

The studies involving human participants were reviewed and approved by Shenzhen People's Hospital, the Second Affiliated Clinical Medical College of Jinan University, the First Affiliated Hospital of Southern University of Science and Technology, China. The patients/participants provided their written informed consent to participate in this study. The animal study was reviewed and approved by Shenzhen People's Hospital, the Second Affiliated Clinical Medical College of Jinan University, the First Affiliated Hospital of Southern University of Science and Technology, China.

## Author Contributions

LW and BL designed the experiments. ZX and DZ performed the experiments. ZZ, WL, RS, JY, and DL made significant revisions to the manuscript. All authors read and approved the final manuscript.

## Conflict of Interest

The authors declare that the research was conducted in the absence of any commercial or financial relationships that could be construed as a potential conflict of interest.
